# Sildenafil Protects against Myocardial Ischemia-Reperfusion Injury Following Cardiac Arrest in a Porcine Model: Possible Role of the Renin-Angiotensin System

**DOI:** 10.3390/ijms161126010

**Published:** 2015-11-12

**Authors:** Guoxing Wang, Qian Zhang, Wei Yuan, Junyuan Wu, Chunsheng Li

**Affiliations:** 1 Department of Emergency Medicine, Beijing Chao-Yang Hospital, Capital Medical University, 8^#^ Worker’s Stadium South Road, Chao-Yang District, Beijing 100020, China; wangguoxing@ccmu.edu.cn (G.W.); zqian604@163.com (Q.Z.); 2000ywei@sina.com (W.Y.); wu007838@sina.com (J.W.); 2 Department of Emergency Medicine, Beijing Friendship Hospital, Capital Medical University, 95^#^ Yong’an Road, Xicheng District, Beijing 100050, China

**Keywords:** sildenafil, myocardial ischemia, renin-angiotensin system, porcine model, Ang (1–7)

## Abstract

Sildenafil, a phosphodiesterase-5 inhibitor sold as Viagra, is a cardioprotector against myocardial ischemia/reperfusion (I/R) injury. Our study explored whether sildenafil protects against I/R-induced damage in a porcine cardiac arrest and resuscitation (CAR) model via modulating the renin-angiotensin system. Male pigs were randomly divided to three groups: Sham group, Saline group, and sildenafil (0.5 mg/kg) group. Thirty min after drug infusion, ventricular fibrillation (8 min) and cardiopulmonary resuscitation (up to 30 min) was conducted in these animals. We found that sildenafil ameliorated the reduced cardiac function and improved the 24-h survival rate in this model. Sildenafil partly attenuated the increases of plasma angiotensin II (Ang II) and Ang (1–7) levels after CAR. Sildenafil also decreased apoptosis and Ang II expression in myocardium. The increases of expression of angiotensin-converting-enzyme (ACE), ACE2, Ang II type 1 receptor (AT1R), and the Ang (1–7) receptor Mas in myocardial tissue were enhanced after CAR. Sildenafil suppressed AT1R up-regulation, but had no effect on ACE, ACE2, and Mas expression. Sildenafilfurther boosted the upregulation of endothelial nitric oxide synthase (eNOS), cyclic guanosine monophosphate (cGMP) and inducible nitric oxide synthase(iNOS). Collectively, our results suggest that cardioprotection of sildenafil in CAR model is accompanied by an inhibition of Ang II-AT1R axis activation.

## 1. Introduction

Cardiac arrest (CA) is a major public health problem cause for both substantial morbidityand mortality among hospitalized patients [1]. Cardiopulmonary resuscitation (CPR) is a critical part of the management of CA. However, myocardial tissue suffers from ischemia/reperfusion (I/R) injury even after successful CPR. Therefore, post-cardiac arrest syndrome is a serious threat to patients, and understanding the underlying cellular and molecular mechanisms may help to develop strategies to treat post-resuscitation myocardial dysfunction.

Renin-angiotensin system (RAS) is one of the major hormone system regulating blood pressure [2]. The RAS is a complex endocrine, paracrine, and autocrine system with many components [2]. Angiotensin I (Ang I) is transformed to Ang II by the angiotensin-converting-enzyme (ACE). Ang II type 1 receptor (AT1R) is one of the major Ang II receptors. Ang (1–7), a recently described heptapeptide product of Ang I and Ang II with biological activity, is formed by ACE2. The endogenous receptor of Ang (1–7) is Mas. In the myocardial system, the Ang II-AT1R axis acts as a detrimental effector by promoting cardiac inflammation and fibrosis [3], causing myocardial cell death and apoptosis [4], whereas the Ang (1–7)-Mas cascade inhibits Ang II expression, thereby providing a cardioprotective effect [5].

Sildenafil, a potent and competitive inhibitor of phosphodiesterase-5 (PDE5) sold as Viagra, is used to treat erectile dysfunction primarily [6,7]. Sildenafil was found to improve exercise hemodynamics, oxygen uptake, heart failure, and pulmonary hypertension [8]. We and other groups have demonstrated the cardioprotection of sildenafil in animal models and human [8,9,10,11,12,13,14]. The major molecular mechanisms include enhancement of nitric oxide-cyclic guanosine monophosphate (cGMP) [15], ERK phosphorylation [16], protein kinase C (PKC) [17], RhoA/ROCK pathways [18], and adrenergic signaling [14]. A recent trial showed that although sildenafil failed to reduce filling pressure in patients with myocardial infarction, it produced beneficial hemodynamic effects on secondary end points in these patients, including enhancing cardiac output (CO), diastolic blood pressure and resistance of vascular vessels [19]. The cardioprotective effect of sildenafil was well-documented in local myocardial ischemic models with left coronary artery ligation [20]. However, whether sildenafil protects myocardial tissue against the stress of global ischemia has not been addressed. There are significant differences between the myocardial ischemic injury induced by coronary artery ligation and CA-resuscitation (CAR). Coronary artery ligation always induces local myocardial ischemia injury whereas CAR can induce global hypoperfusion that results in damage in other organs, such as cerebral I/R injury [21] and kidney I/R injury [22].

This work examined the effects of sildenafil infusion on the survival rate and cardiac indexes following global ischemia by inducible CA. Moreover, we firstly observed the influence of sildenafil infusion on the RAS system including plasma Ang (1–7) and Ang II levels. At last, the expression of ACE, AT1R, ACE2, and Mas in sildenafil-infused myocardial tissue were evaluated.

## 2. Results

### 2.1. Sildenafil Improves Survival Rate in Pig CAR Model

**Figure 1:**
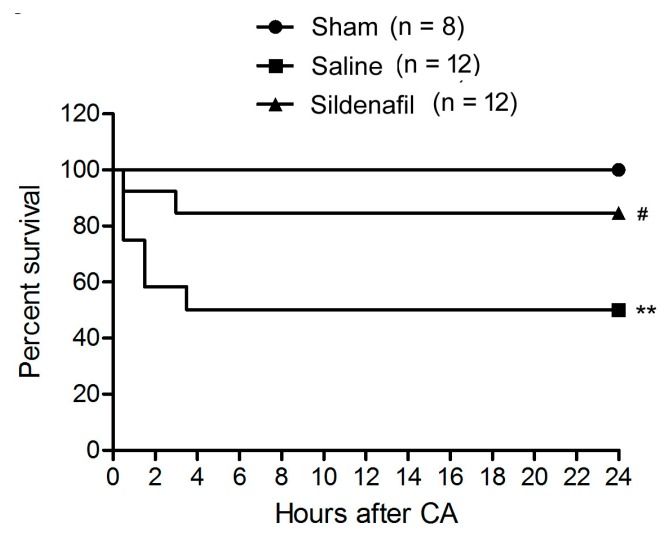
Survival curve of animals in the present study. ** *p* < 0.01 *vs.* Sham, **^#^** *p* < 0.05 *vs.* Saline.

We did not detect any significant differences of baseline hemodynamic parameters in the three groups (data not shown). During the post-resuscitation, the number of defibrillation shocks in sildenafil-treated pigs was significantly less compared to saline-treated pigs (2.1 ± 0.6 *vs*. 4.9 ± 1.2, *p* < 0.05, Table 1). The energy of shock and time to Restoration of Spontaneous Circulation (ROSC) in sildenafil-treated pigs was also lower compared to the values in saline-treated pigs (Table 1). At 24 h after ROSC, there were six surviving pigs in the Saline group, whereas there were ten surviving pigs in the sildenafil-treated group (Table 1). Figure 1 illustrated the survival curve.

**Table 1:** Outcome measures after post-resuscitation in sham, saline, and sildenafil-treated groups in a porcine myocardial ischemia/reperfusion (I/R) injury model.

Outcomes	Sham (*n* = 8)	Saline (*n* = 12)	Sildenafil (*n* = 12)
Number of shocks	0	4.9 ± 1.2 **	2.1 ± 0.6 ^#,^**
Total adrenaline dose (mg)	0	1.9 ± 0.7 **	0.8 ± 0.3 ^#,^**
Energy of shock (J)	0	260.5 ± 27.8 **	212.7 ± 24.2 ^#,^**
Time to ROSC (min)	0	6 ± 2.2 **	4 ± 1.3 **
Number of surviving pigs (at ROSC)	8	9	11
Number of surviving pigs (6-h after ROSC)	8	6 **	10 ^#^

Values are means ± SD or numbers (*n*). ROSC = restoration of spontaneous circulation. ** *p* < 0.01 *vs.* sham, ^#^ *p* < 0.05 *vs.* saline.

### 2.2. Sildenafil Ameliorates the Reduced Cardiac Function in Pig CAR Model

**Figure 2:**
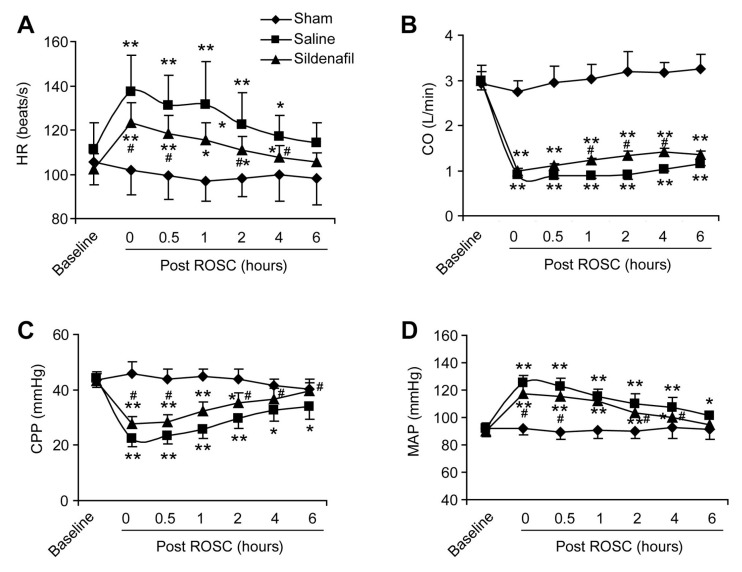
Cardiac functions of pigs after post-resuscitation in sham, saline, and sildenafil-treated groups. The heart rate (**A**) HR (beats/s), cardiac output (**B**) CO (L/min), coronary perfusion pressure (**C**) CPP (mmHg), and mean aortic pressure (**D**) MAP (mmHg) were monitored at 0, 0.5, 1, 2, 4, and 6 h after ROSC. * *p* < 0.05 *vs.* Sham, ** *p* < 0.01 *vs.* Sham, **^#^** *p* < 0.05 *vs.* Saline. *n* = 6–10 per group.

The HR of pigs experiencing post-resuscitation significantly increased after ROSC (*p* < 0.05, Figure 2A). However, sildenafil pretreatment significantly inhibited this increase of HR (at 0.5, 2 and 4 h post ROSC, Figure 2A). CO was also suppressed in the Saline group, but was partly but significantly elevated by sildenafil pretreatment (at 1, 2 and 4 h post ROSC, Figure 2B). CPP was reduced by post-resuscitation, while sildenafil significantly rescued CPP (Figure 2C). CA and resuscitation boosted the MAP and sildenafil further decreased the MAP significantly at 30 min, 2 h, and 4 h post ROSC (Figure 2D). These results suggest that sildenafil ameliorates the reduced cardiac function in pig CAR model.

### 2.3. Plasma Ang II and Ang (1–7) Levels Are Decreased by Sildenafil in Pig CAR Model

**Figure 3:**
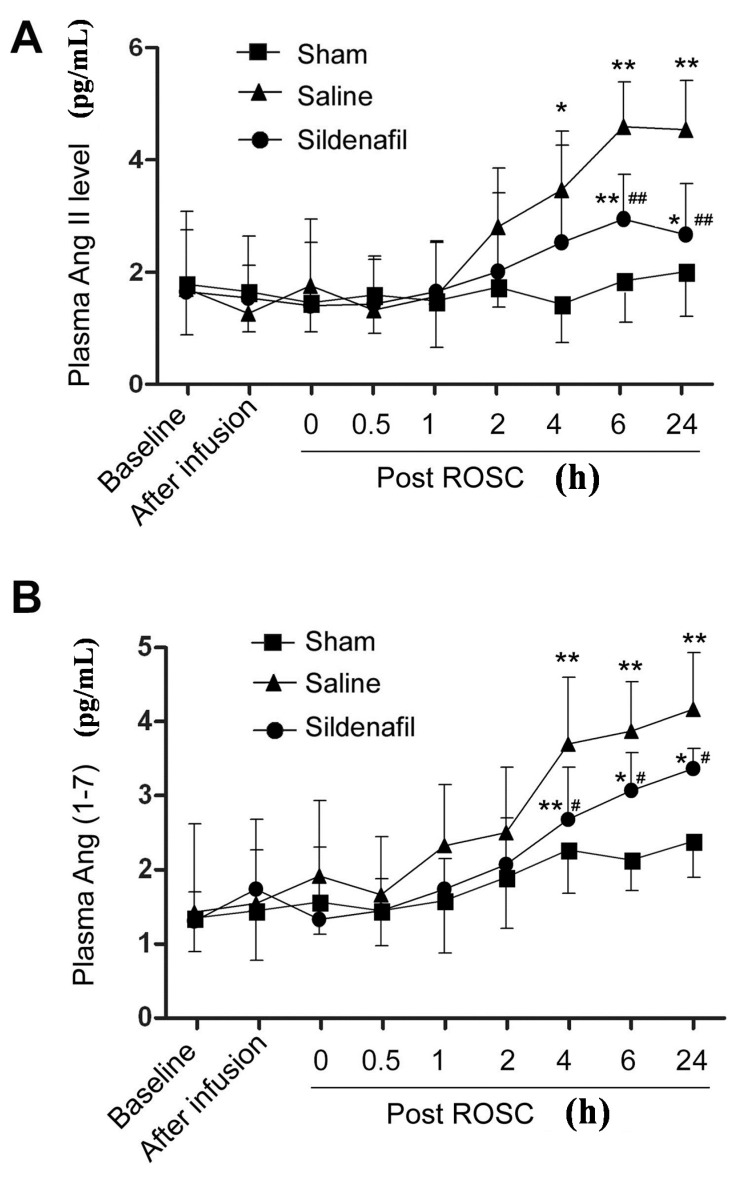
Plasma levels of the RAS components Ang II and Ang (1–7) in a porcine myocardial I/R injury model. ELISA determination of Ang II (**A**) and Ang (1–7) (**B**) at 0, 0.5, 1, 2, 4, 6 and 24 h in sham, saline, and sildenafil-treated groups following restoration of spontaneous circulation (post ROSC, h) were determined by ELISA. * *p* < 0.05 *vs.* Sham, ** *p* < 0.01 *vs.* Sham, **^#^** *p* < 0.05 *vs.* Saline, **^##^** *p* < 0.01 *vs* Saline *n* = 6–10 per group.

When plasma Ang II levels of pigs from the three treatment groups were examined, the Ang II levels at 4, 6 and 24 h post ROSC markedly increased (Figure 3A). Sildenafil pretreatment successfully reduced the upregulation of plasma Ang II compared to the Saline group. Significant sildenafil-induced reductions in Ang II levels were observed at 6 and 24 h post ROSC (Figure 3A). A similar result was observed for plasma Ang (1–7) levels (Figure 3B), which were elevated post-resuscitation from 1 to 24 h post ROSC. Elevated Ang (1–7) levels in the Saline group were partly decreased by sildenafil pretreatment. Significant sildenafil-induced reductions in Ang (1–7) serum levels were measured at 4, 6 and 24 h post ROSC (Figure 3B).

### 2.4. Sildenafil Attenuates Myocardial Apoptosis in Pig CAR Model

**Figure 4:**
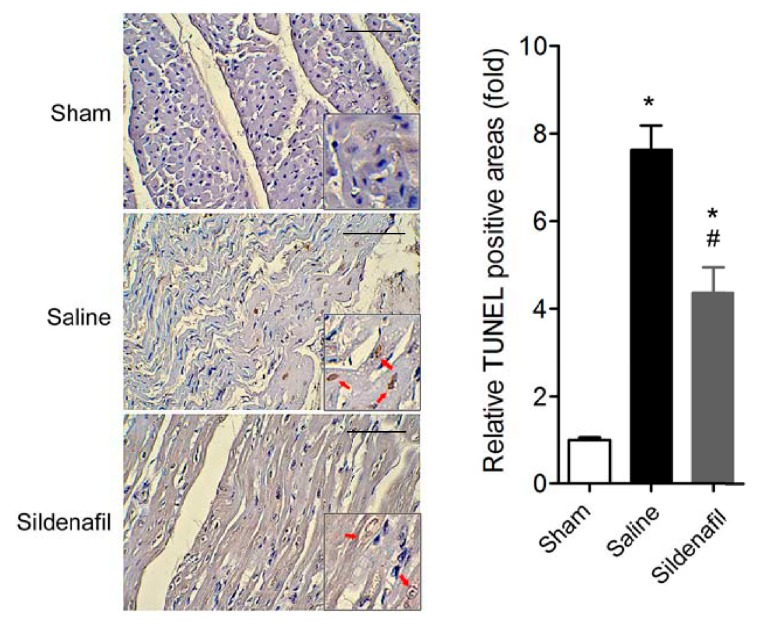
Myocardium apoptosis was assayed by TUNEL. Representative image and quantitative analysis of TUNEL staining on myocardium. * *p* < 0.05 *vs.* Sham, ^#^ *p* < 0.05 *vs.* Saline. *n* = 6–10 per group. Scale bar: 100 μm. The red arrows indicate the TUNEL-positive nuclei.

As shown in Figure 4, TUNEL assay showed that CAR injury induced remarkable apoptosis in myocardium. However, sildenafil treatment partly attenuated the myocardial apoptosis.

### 2.5. Sildenafil Reduces Myocardial Ang II Expression in Pig CAR Model

**Figure 5:**
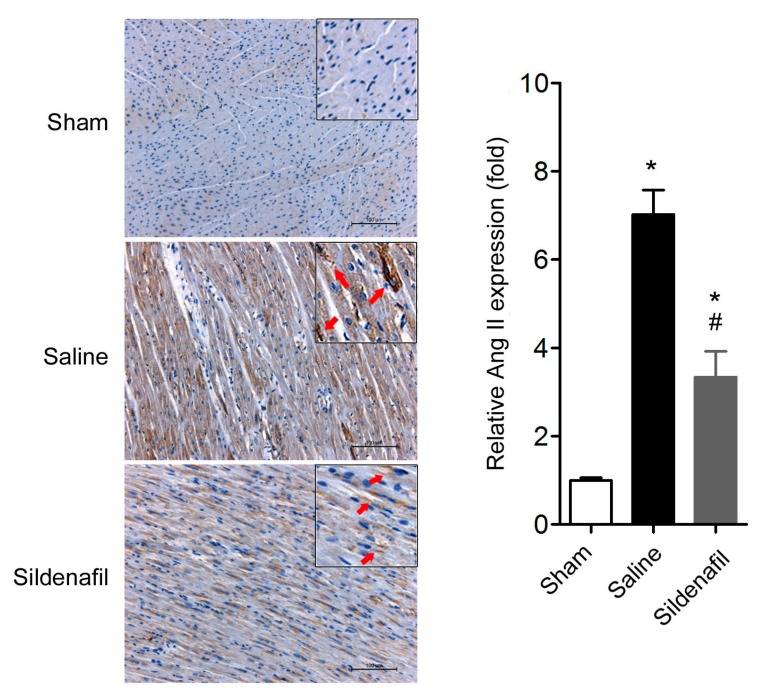
Myocardial Ang II expression was determined by immunohistochemistry. Representative image and quantitative analysis of Ang II immunohistochemical staining in myocardium. * *p* < 0.05 *vs.* Sham, ^#^ *p* < 0.05 *vs.* Saline. *n* = 6–10 per group. Scale bar: 100 um. The red arrows indicate the positive expression of Ang-II.

We also evaluated the myocardial Ang II expression using immunohistochemistry. As shown in Figure 5, myocardial Ang II expression was significantly increased after ROSC in this model. Sildenafil treatment partly decreased the myocardial Ang II upregulation.

### 2.6. Sildenafil Does not Change ACE and ACE2 Expressions in Heart Tissue in Pig CAR Model

**Figure 6:**
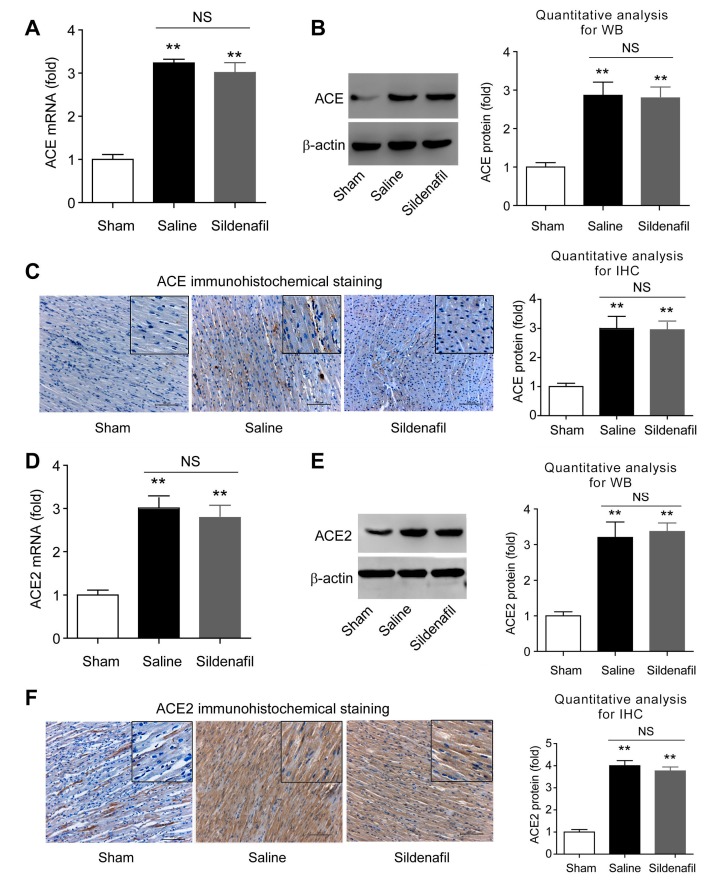
Tissue mRNA and protein levels of ACE and ACE2 in myocardial tissue after post-resuscitation in a porcine myocardial I/R injury model. Effects of sildenafil on ACE expression were assayed by qRT-PCR (**A**), Western blotting (**B**), and immunohistochemistry (**C**), and compared to the sham and saline-treated groups. ** *p* < 0.01 *vs*. Sham; NS, no significance. *n* = 6–10 per group. Effects of sildenafil on ACE2 expression were measured by qRT-PCR (**D**), Western blotting (**E**), and Immunohistochemistry (**F**), and compared to the sham and saline-treated groups. ** *p* < 0.01 *vs*. Sham; NS, no significance. *n* = 6–10 per group. Scale bar in **C**,**F**: 100 μm.

Next, the expression of ACE and ACE2 proteinin the heart were studied. ACE mRNA level in pigs experiencing post-resuscitation (Saline group) were significantly higher (Figure 6A). Immunoblotting and immunohistochemistry analyses confirmed that ACE was increased on the protein level by post-resuscitation (Figure 6B,C). However, there were no significant differences of cardiac ACE expression between saline and sildenafil-treated pigs (Figure 6A–C). Similarly, no differences in ACE2 mRNA (Figure 6D) and protein (Figure 6E,F) levels were observed. These data indicate that sildenafil is unable to change ACE and ACE2 protein expression in porcine heart tissue after post-resuscitation.

### 2.7. Sildenafil Decreases AT1R But Has no Effect on Mas Expression in Pig CAR Model

**Figure 7:**
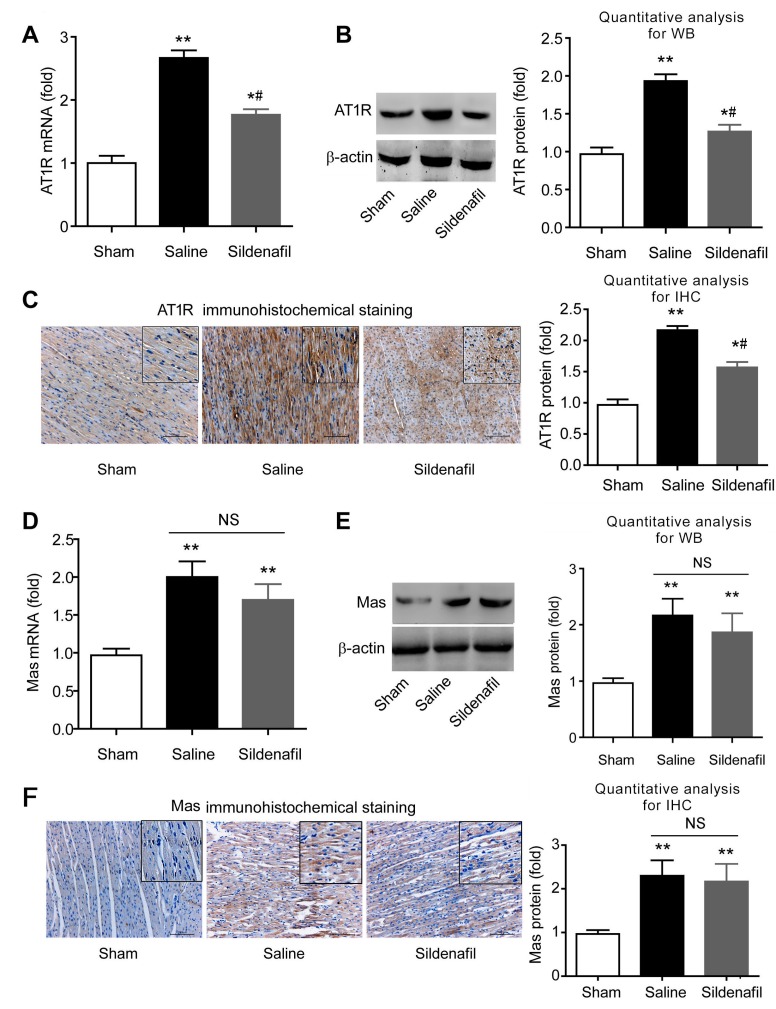
The expression of AT1R and Mas (mRNA and protein) after post-resuscitation in a porcine myocardial I/R injury model. Effects of sildenafil on AT1R mRNA level and protein levels were determined by qRT-PCR (**A**), Western blotting (**B**), and Immunohistochemistry (**C**) and compared to the sham and saline-treated groups. * *p* < 0.05 *vs*. Sham, ** *p* < 0.01 *vs*. Sham; ^#^ *p* < 0.05 *vs*. Saline. NS, no significance. *n* = 6–10 per group. Effects of sildenafil on Mas expression were measured by qRT-PCR (**D**), Western blotting (**E**), and Immunohistochemistry (**F**), and compared to the sham and saline-treated groups. * *p* < 0.05 *vs*. Sham, ** *p* < 0.01 *vs*. Sham; ^#^ *p* < 0.05 *vs*. Saline. NS, no significance. *n* = 6–10 per group. Scale bar in **C**,**F**: 100 μm.

Expression of AT1R and Mas were determined. Post-resuscitation induced AT1R mRNA (Figure 7A) and protein (Figure 7B,C) expression in myocardial tissue. Sildenafil partly, but significantly blocked these effects (Figure 7A–C). Similarly, Mas mRNA (Figure 7D) and protein (Figure 7E,F) expression were increased following post-resuscitation in myocardial tissue. However, sildenafil had no significant effect on the post-resuscitation induced Mas expression in porcine hearts (Figure 7D–F).

### 2.8. Sildenafil Increases eNOS, iNOS and cGMP Levels in Myocardial Tissue in Pig CAR Model

**Figure 8:**
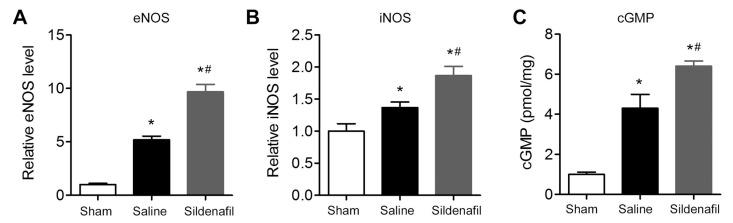
Effect of sildenafil on the levels of endothelial nitric oxide synthase (eNOS), inducible nitric oxide synthase (iNOS), and cyclic guanosine monophosphate (cGMP) in myocardial tissue after post-resuscitation in a porcine myocardial I/R injury model. (**A**) Relative eNOS levels; (**B**) relative iNOS levels; and (**C**) relative cGMP levels in myocardial tissue from sham, saline, and sildenafil-treated groups. A total of 20 μg of cardiac protein homogenate was used to measure the levels of eNOS, iNOS, and cGMP by ELISA. The levels of eNOS, iNOS, and cGMP in the Sham group were set as 1 and the relative fold increase in tissue of the saline and sildenafil group was calculated, respectively. * *p* < 0.05 *vs*. Sham; ^#^ *p* < 0.05 *vs*. Saline. *n* = 6–10 per group.

Finally, the levels of eNOS, iNOS, and cGMP in porcine myocardial tissue were measured in the myocardial I/R injury model. Levels of eNOS, iNOS, and GMP were significantly up-regulated in myocardial tissue in pig CAR model (Figure 8A–C). Sildenafil pretreatment further increased eNOS, iNOS, and cGMP levels significantly in porcine myocardial tissue (Figure 8A–C).

## 3. Discussion

Erectile dysfunction in men is the original indication of sildenafil [6]. Sildenafil inhibits PDE5 to increase intracellular cGMP concentrations in the myocardium, leading to relaxation of vascular smooth cell contraction. All these events finally result in the enhancement of blood flow in tissues [6]. This hemodynamic feature prompted researchers to explore sildenafil’s potential beneficial effects on the heart. The cardioprotective effects of sildenafil have been widely confirmed in various ischemic diseases [9,16]. Moreover, in other myocardial dysfunction, sildenafil also displayed potent cardiprotection. Isidori AM *et al.* [8,14] showed that a cGMP-dependent activation of PDE2 was induced by sildenafil with a pronounced cardioprotection. Giannetta, *et al.* [12,13] showed that sildenafil treatment improved cardiac kinetics and blood biomarkers in human with a good safety profile. In the present study, a cardioprotective role of sildenafil in post-cardiac arrest syndrome via modulating RAS was demonstrated. First, sildenafil was able to decrease the number of shocks necessary for resuscitation and it enhanced the survival rate in a porcine myocardial ischemia model induced by post-resuscitation. The cardiac function of pigs after post-resuscitation was significantly improved by sildenafil pretreatment; Second, the potential relationship between sildenafil and RAS activation during post-resuscitation was investigated. Myocardial ischemia induced by post-resuscitation remarkably increased serum Ang II and Ang (1–7) level. Post-resuscitation also elevated local expression of ACE, ACE2, AT1R, and Mas in myocardial tissue. Interestingly, sildenafil did not alter ACE, ACE2, and Mas levels in myocardial tissue, but attenuated the elevations of plasma Ang II, Ang (1–7), and myocardial AT1R expression; At last, sildenafil further increased the eNOS, iNOS and cGMP levels in myocardial tissue. All these results indicate that the modulation of the RAS cascade may contribute to the protective effect of sildenafil in post-resuscitation.

To our knowledge, this is the first *in vivo* study showing that sildenafil pretreatment alters RAS activation after CA, which raises the possibility that sildenafil protects myocardial I/R injury through modulating the RAS. Systemic or local RAS activation represents a critical step for chronic ventricular remodeling, which is an important determinant for prognosis after myocardial infarction [23]. AT1R mRNA was up-regulated 6.6-fold in myocytes from the left ventricle after myocardial infarction [24]. Inhibition of ACE or blockade of AT1R reduced apoptosis [25] and inhibited myocardial remodeling after myocardial infarction in pigs [26]. Accordingly, AT1R knockout mice with myocardial infarction displayed less myocardial remodeling and improved survival [27]. Conversely, Ang (1–7) attenuated heart failure procedure [28], while a non-peptide Mas agonist, AVE-0991, largely prevented myocardial infarction-associated heart failure [29]. These studies collectively suggested the detrimental effect of the Ang II-AT1R axis, as well as the favorable effect of the Ang (1–7)-Mas axis in the pathophysiological process after myocardial infarction. It should be noted that these effects were typically observed two or more days post myocardial infarction. Plasma Ang II and Ang (1–7) level were acutely triggered at 1–2 h post ROSC, while Ang II and Ang (1–7) level were attenuated by sildenafil pretreatment. Moreover, sildenafil inhibited the upregulation of Ang II and AT1R in myocardial tissue, but had no effect on expression of Mas, ACE, and ACE2. The elevation of Ang (1–7) might be a quick counteractive response against the enhancement of plasma Ang II and thereby exhibiting cardioprotection. Sildenafil successfully compromised the elevation of cardiac/plasma Ang II and cardiac AT1R expression, suggesting that sildenafil may partly block the detrimental effect of post-ischemic RAS activation. Unexpectedly, sildenafil pretreatment also suppressed the enhancement of plasma Ang (1–7) levels, while cardiac Mas expression was not altered by sildenafil. Although it is not known how sildenafil decreases Ang (1–7) levels, these results could rule out the possibility that Ang (1–7)-Mas cascade plays critical roles in cardioprotection of sildenafil. We, therefore, propose that sildenafil protects myocardial I/R injury via inhibiting Ang II-AT1R axis, but not by strengthening the Ang (1–7)-Mas axis. Dias *et al.* reported that sildenafil reduced Ang II expression in kidney rather than plasma Ang II [30]. We think this difference may be due to the discrepancy between our CAR model and their hypertensive model. Furthermore, Straubinger J *et al.* reported that they found higher cGMP levels in cultured cardiomyocytes and AT1R overexpression upregulated the cGMP-dependent protein kinase type I [31]. However, they also found that the progressive cardiomyocytes hypotrophy and fibrosis was not prevented by prolonged sildenafil treatment in the AT1R heart-specific transgenic mice model [31]. This discrepancy may be due to the difference between species.

The enhancement of eNOS, iNOS, and cGMP are believed to be major mechanisms of action for sildenafil [16,32] and other cardioprotectors, such as bradykinin [33]. However, this viewpoint was challenged by some investigations with opposing findings. Elrod *et al.* reported that utilization of sildenafil did not alter myocardial cGMP levels [34]. Recent studies have revealed additional molecules involved in the cardioprotective effect of sildenafil. These molecules include SIRT1 [35], protein kinase C [17], and phospholemman [36]. In the present study, cardiac ischemia increased eNOS, iNOS, and cGMP levels at 24 h post ROSC, and were further enhanced by sildenafil treatment. Furthermore, the energy required to return the pig to spontaneous circulation was much less in the sildenafil treated pigs and time to ROSC was also lower. We considered that the increased NO production by sildenafil may increase blood supply to myocardium, which thereby reduces the sympathetic tone and heart rate. So it is easier to defibrillate in the sildenafil-treated pigs. It should be noted that Garcia LA and Venneri MA demonstrated that sildenafil decreased iNOS expression in pelvic ganglia neurons and streptozotocin-induced diabetic mice model respectively [37,38]. We think this discrepancy might be due to the differences of experimental models. Collectively, these results support the notion that eNOS, iNOS, and cGMP elevation and the enhancement of NO production may contribute to the cardioprotection of sildenafil.

Moreover, I/R injury induces several pathophysiological changes, including reduced NO bioavailability [39], calpain activation [40] and energy status [41]. The anti-apoptosis effect of sildenafil may be another molecular mechanism for its cardioprotection. Sildenafil was reported to inhibit apoptosis and necrosis via NO signaling in cultured myocytes or in a chronic model of doxorubicin cardiotoxicity [11,15]. In our study, we found sildenafil attenuated TUNEL-positive cells in myocardium, supporting the anti-apoptosis action of sildenafil in myocardial I/R injury.

In fact, NO has been reported to inhibit rat aortic smooth muscle cell migration driven by Ang II via blocking AT1R [42]. Moreover, NO inhibits Ang II-induced activation of the calcium-sensitive tyrosinekinase proline-rich tyrosine kinase 2 in cardiac fibroblasts [43]. In this study, we found that sildenafil upregulated eNOS, iNOS and cGMP. We speculate that these upregulation would result in increased NO level and thereby inhibit Ang II-AT1R axis. These results raise an interesting question of how sildenafil down-regulates myocardial AT1R. Cahill *et al.* reported that chronic treatment of cells with a group of agents with nitric oxide (NO)-generating activity could decrease AT1R activity in dose- and time-dependent manners [44]. Another study showed that *S*-nitroso acetyl dl-penicillamine, a potent NO donor, suppressed the expression level of AT1R mRNA by 90% and AT1R number by 60% after 24 h of stimulation [45]. Conversely, treatment with an NO synthase inhibitor up-regulated the expression of the AT1R in an adrenal glomerulosa cell line [46]. NO was also showed to reduce AT1R protein expression in neurons [47]. However, there are controversial results on whether cGMP is important for the regulation of AT1R by NO. NO-induced down-regulation of AT1R is independent of cGMP in vascular muscle cells [46], but is cGMP-dependent in neurons [47]. Given that sildenafil is able to increase NO production through up-regulating eNOS and iNOS, it is safe to speculate that sildenafil down-regulates AT1R in the heart via inducing eNOS and iNOS. Whether cGMP participates in this effect of sildenafil needs further investigation.

In a previous work by our group [48], the major object was to investigate the action of sildenafil on apoptotic signaling pathway, microRNAs expression and nitric oxide syntheses levels. In that study, we found that sildenafil reduced TUNEL-positive cell number, enhanced the ratio of Bcl-2/Bax and prohibited activity of caspase-3 in myocardial tissues. Additionally, sildenafil treatment inhibited the increases in the microRNA-1 levels and alleviated the decreases in the microRNA-133a levels which negatively regulate pro-apoptotic genes [48]. In this study, we focused on the Ang II-AT1R axis. Sildenafil partly attenuated the increases of plasma angiotensin II (Ang II) and Ang (1–7) levels after CAR. Sildenafil also decreased apoptosis and Ang II expression and AT1R upregulation in myocardium. We considered that these results were novel compared with our previous publication [48] and may add new information on the cardioprotection of sildenafil.

There are several limitations of this study. First, in the porcine myocardial ischemia/reperfusion (I/R) injury model for CA healthy pigs were used, whereas most humans suffering from CA are not healthy. Pathophysiological dysfunction, such as hyperglycemia and hyperlipemia, always exist in individuals suffering from CA. These malfunctions may affect the outcome of sildenafil treatment in humans; Second, the inducible CA in the present study was achieved by acute VF, which may not reflect some chronic myocardial pathological changes in humans; Third, sildenafil was administrated before CA to illustrate the protective effect of sildenafil against global ischemic injury rather than limited myocardial tissue ischemia; Finally, NO inhibitors to block the increase of NO signaling were not used in this study. Whether sildenafil would still be effective in such a setting may give an answer for the requirement of NO signaling for the cardioprotection of sildenafil.

## 4. Materials and Methods

### 4.1. Animals

Inbred male landrance miniature piglets (11–13 months old, 30 ± 2 kg) were used as described previously [49]. All animals were housed in a controlled environment and fed with standard chow. All procedures were conducted in compliance with guidelines of Animal Care and Use Committee of the Capital Medical University. The experimental protocol was also approved by the Animal Experiments Committee of the Capital Medical University (permit number: 2010-D-013).

### 4.2. Experimental Procedures

Anesthesia was achieved in the animals. Midazolam was intramuscularly injected (0.5 mg/kg) and followed with ear vein injection of propofol (1.0 mg/kg). Then, all the animals were fixed in a surgical plane of anesthesia. Sodium pentobarbital (8 mg/kg per hour) were intravenously infused to maintain anesthesia. An endotracheal tube (cuffed 6.5-mm) was inserted into trachea and a volume-controlled ventilator (Servo 900C; Siemens, Munich, Germany) was used. The tidal volume was set at 12 mL/kg and the respiratory frequency was set at 12 breaths/min. An inline infrared cacographic (RespirometricInc, Murrysville, PA, USA) was used to monitor end-tidal pCO_2_. To maintain respiratory frequency, the end-tidal pCO_2_ was kept between 35 and 40 mmHg. During the procedure, acetated Ringer’s solution was infused for compensating the fluid losses during the first hour of preparation. After that, acetated Ringer’s solution was infused at 20 mL/kg and glucose-electrolytes solution (2.5% 8 mL/kg per h) was added.

To monitor aortic pressure and get blood samples, an angiographic catheter was inserted from the right femoral artery into the aortic arch. To monitor CO, a 7 FrSwan-Ganz catheter (Edwards Life Sciences, Irvine, CA, USA) was inserted from the right femoral vein and flow-directed into the pulmonary artery [50]. The electrocardiogram and hemodynamic parameters measured by OmniCare M1165/66A system (Hewlett Packard, Andover, MA, USA). Self-adhesive defibrillation electrodes were located on the chest wall. CPP was calculated by subtracting the right atrial from the aortic diastolic pressure.

### 4.3. Experimental Myocardial I/R Injury by CA

Experimental myocardial I/R injury by CA in pigs was induced as described previously [49]. The pigs were randomly divided into three groups: Sham group (*n* = 8), Saline (0.9% NaCl) group (*n* = 12), and Sildenafil group (*n* = 12). Sildenafil was obtained from commercially available tablets (Viagra^®^, 25 mg tablet, Pfizer Inc., New York, NY, USA) and dissolved with 50 mL saline. After the above mentioned preparations, animals were infused with the sildenafil solution (0.5 mg/kg) or the same volume saline intraperitoneally 30 min prior to VF and subjected with equilibration for 30 min. Baseline measurements and blood were obtained during this time. The temporary pacemaker conductor was inserted into the right ventricle through the right internal jugular vein and connected to a GY-600A-model electrical stimulator (Kaifeng Huanan Equipment Co., Ltd., Kaifeng, China), which was used to induce ventricular fibrillation (VF). The model of electric current was set at as follows: 300/200 ms, 40 V, 8:1 proportion, 10 ms step length). Once VF was induced, the mean aortic pressure was rapidly decreased to zero [51]. Meanwhile, the mechanical ventilation was discontinued. External biphasic wave form defibrillation beginning at 3 J/kg was delivered to attain ROSC 8 min later. If the initial defibrillation shock failed, energy was increased with 1 J/kg increments. Cardiopulmonary resuscitation (CPR) was performed when the defibrillation and ROSC failed. Also, manual chest compressions were conducted at a rate of 100 compressions per minute and lasted for 2 min. Then, another defibrillation attempt was achieved. If the first defibrillation was unsuccessful, epinephrine (0.02 mg/kg) was swiftly injected intravenously. After this, two min of CPR was performed. Ventilation was also used (compression-to-ventilation ratio, 30:2) [52]. If spontaneous circulation cannot be made, the animals would subjected with CPR for more two minutes and defibrillation was conducted once more.

We defined ROSC as the return of a palpable pulse with a systolic blood pressure of >50 mm Hg. The animal was believed to be dead if the spontaneous circulation was not restored within 0.5 h [53]. The animals were ventilated with 100% oxygen after ROSC. The animals were allowed to recover from anesthesia. After that, the pigs were tightly monitored for additional 18 h. During this period, blood samples were obtained at 0 min, 0.5, 2, 4 and 6 h after ROSC.

During the 6h intensive care period post inducible global ventricular fibrillation and cardiopulmonary resuscitation, we monitored the HR, CO, CPP and MAP in these animals hourly. We evaluate the health of pigs by Cerebral Performance Category (CPC) score [54]. If the CPC score >3, we would perform euthanasia in the animal using intravenous injection of barbiturates. In this period, three pigs in saline-treated group and 1 pig in sildenafil-treated group died due to sudden cardiac arrest. We performed standard cardiopulmonary resuscitation on these animals but failed. It should be noted that the swine Cerebral Performance Category (CPC) score [54] remained 1–2 before sudden death. None of other pigs in the three groups died during 6 to 24 h. At last, the animals were rapidly anaesthetized by a bolus of intravenous propofol (100 mg) and potassium chloride (10%, 10 mL). The apex of left ventricular cardiac tissue was isolated, immediately frozen in liquid nitrogen and stored at −80 °C.

### 4.4. Hemodynamic Parameters

HR, CO, CPP, and MAP at six time-points (0, 0.5, 1, 2, 4, and 6 h) after ROSC were recorded. MAP was monitored via the right femoral arterial catheter.

### 4.5. Enzyme-Linked Immunosorbent Assay (ELISA)

The protein levels of Ang II, eNOS, and iNOS in blood plasma or cardiac tissue were measured using commercial ELISA kits (BlueGene Biotech Co., Ltd., Shanghai, China). The plasma level of Ang (1–7) was determined by an ELISA kit from BlueGene Biotech Co., Ltd. (Shanghai, China). Briefly, 50 μL blood plasma or 20 μg protein homogenate of cardiac tissue was added into a 96-well plate and ELISAs were performed according to the manufacturer’s instructions. Cardiac tissue was homogenized with PBS plus protease inhibitor. OD values (450 nm) were determined in a microplate reader (Tecan M200, Munich, Germany) as previously described [55].

### 4.6. Quantitative Real-Time PCR (qRT-PCR)

The total RNA was isolated from heart tissue using Trizol (Invitrogen, Carlsbad, CA, USA) and reverse transcribed to single cDNA [56]. The cDNA was subjected to qRT-PCR with ABI 7500 system (Applied Biosystems, Foster City, CA, USA) using the following specific primers: ACE (forward: 5′-ATC AAG CGG ATC ATA AAG AAG-3′, reverse: 5′-CAC GCT GTA GGT GGT TTC C-3′); ACE2 (forward: 5′-TCT GAA TGA CAA CAG CCT AG-3′, reverse: 5′-CAC TCC CAT CAC AAC TCC-3′); Mas (forward: 5′-TAT TCC TCA TCT TCG CTA T-3′, reverse: 5′-GCC CTG GTC AGA ACA ACT-3′); AT1R (forward: 5′-TCA CCT GCA TCA TCA TCT GG-3′, reverse: 5′-AGC TGG TAA GAA TGA TTA GG-3′), and GAPDH (forward: 5′-GAC CCA GAA TAC CAA GTG CAG ATG TA-3′; reverse: 5′-CTG TTT CAG GAT TTA AGG TTG GAG ATT-3′). Gene expression was normalized to GAPDH. Data analysis was performed using the 2^−∆∆*C*t^ method [57].

### 4.7. Immunohistochemical Staining

Immunohistochemical assay in heart tissue was performed as described previously [58]. Briefly, myocardial tissue sections were placed in histosol to remove the paraffin and then rehydrated in graded ethanol. After blocking in 5% BSA for 4 h, the sections were incubated with following primary antibodies against: ACE2 (1:200; Abcam, Cambridge, UK), ACE (1:200; Abcam), Mas (1:300; Alomone Lab, Jerusalem, Israel), and AT1R (1:200; Abcam) overnight at 4 °C. After being washed with PBS solution (5 min × 3 times), sections were immersed in solutions with secondary antibodies at room temperature for 2 h and followed by avidin-biotin peroxidase solution incubation. Sections stained with normal rabbit serum served as negative control. After PBS washes, the color in sections were developed by diaminobenzidine tetrahydrochloride (DAB, Sigma, Milwaukee, WI, USA) and counter stained by hematoxylin. The images were captured by IX80 microscopy (Olympus, Tokyo, Japan) and analyzed with Image Pro Plus system (Carlsbad, CA, USA). More than 15 images were analyzed for every group.

### 4.8. Western Blot Analysis

Western blot analysis was performed as described previously [59]. After harvesting heart tissue, phosphate-buffered saline (PBS, 0.1 mmol/L) was used to wash it. Then, the tissues were homogenized with the RIPA lysis buffer plus protease inhibitors (Pierce, CA, USA). After measuring the total protein concentration with the BCA assay (Beyotime, Haimen, China), 30 μg samples were run on a 10%–12% SDS gel. The protein was subsequently electrically transferred onto a nitrocellulose membrane and blocked using 5% evaporated milk in PBS. Then, the membranes were incubated with primary antibodies against ACE2 (1:200), ACE (1:200), AT1R (1:300) and Mas (1:300) in TBS-plus buffer solution for 4 h at room temperature. After another set of rinses with PBS, membranes were incubated with HRP-conjugated secondary antibodies for 2 h. HRP signals were detected with a chemiluminescence detection system (Amersham Biosciences, Little Chalfont Bucks, UK).

### 4.9. TUNEL Assay

To detect nuclear DNA fragmentation in apoptosis, TUNEL assay was conducted with a commercial kit (Roche Diagnostics, Mannheim, Germany) as described previously [60].

### 4.10. Measurement of cGMP Levels

A total of 100 mg myocardial tissue from each animal was extracted in 2 mL RIPA lysis buffer. The homogenates were then centrifuged (12,000× *g*, 10 min). The supernatant was collected and aliquoted and stored at −20 °C for cGMP determination. The cGMP levels in the extracted supernatant were assayed using commercial ELISA kit (R&D Systems, Minneapolis, MN, USA). The average optical density was obtained using microplate reader (Tecan M200, Munich, Germany) and the values are calculated with standard curve.

### 4.11. Statistical Analyses

Results are expressed as the means ± SD. Comparisonbetween groups was done using the Student’s *t* test or analysis of variance (ANOVA). To compare the survival rate, Chi-Square analysis was used. *p* < 0.05 was considered statistically significant.

## 5. Conclusions

In summary, the administration of sildenafil improves the survival after post-resuscitation in a porcine CAR myocardial I/R injury model. We also provide the first evidence that the inhibition on Ang II-AT1R axis may contribute to the cardioprotection of sildenafil in CAR model. Our results may add a new perspective to the understanding of the cardioprotective effects of sildenafil.
